# Supplementary material for: Self-reactivity controls functional diversity of naive CD8+ T cells by co-opting tonic type I interferon
Source: Nat Commun. 2021 Oct 18;12:6059. doi: 10.1038/s41467-021-26351-3 (PMC8523551; doi:10.1038/s41467-021-26351-3)
Supplement: Supplementary file 1 — Supplementary Information [file 41467_2021_26351_MOESM1_ESM.pdf]

Supplementary Information

Self-reactivity controls functional diversity of naive $CD8^+$ T cells by co-opting tonic type I interferon

Young-Jun Ju, Sung-Woo Lee, Yoon-Chul Kye, Gil-Woo Lee, Hee-Ok Kim, Cheol-Heui Yun, and Jae-Ho Cho

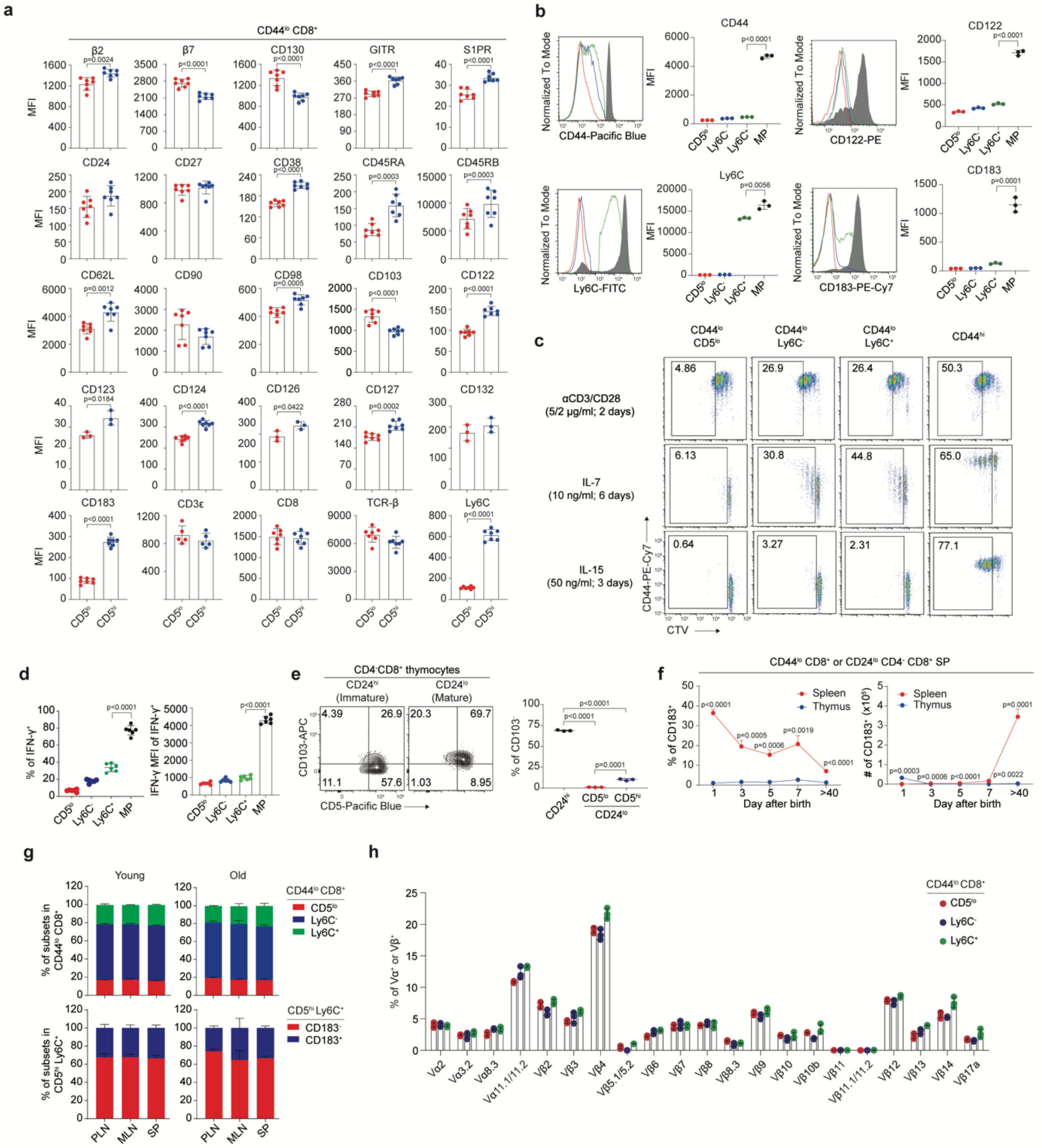

Supplementary Figure 1

Phenotypic heterogeneity of peripheral naive CD8^{+} T cells

a, Expression of various surface molecules on CD5^{lo} and CD5^{hi} B6 naive CD8^{+} T cells (n=7 mice for each group). **b,** Levels of CD44, CD122, Ly6C and CD183 expression in B6 CD8^{+} T cell subsets indicated (n=3 mice for each group). **c,** Proliferation of the indicated subsets of B6 CD8^{+} T cells after stimulation with plate-bound anti-CD3/CD28, IL-7 or IL-15 in vitro. **d,** IFN-

γ production of the indicated subsets of B6 $CD8^+$ T cells after 5h PMA and ionomycin stimulation in vitro (n=6 mice for each group). **e,** Percentage of $CD103^-$ cells in $CD24^{lo}$ $CD5^{lo}$, $CD24^{lo}$ $CD5^{hi}$, and $CD24^{hi}$ $CD4^-CD8^+$ B6 thymocytes (n=3 mice for each group). **f,** Splenic and thymic $CD183^+$ B6 naive $CD8^+$ T cell changes over time after birth (n=3 mice for each group). **g,** Proportions of B6 naive $CD8^+$ T cell subsets in peripheral lymphoid organs of young adult (4-8 wk) and aged (>1 yr) mice (PLN; peripheral lymph node, MLN; mesenteric lymph node, SP; spleen) (n=3 mice for each group). **h,** Expressions of several TCR variable chain repertoire in B6 naive $CD8^+$ T cell subsets (n=3 mice for each group). Statistical significance was confirmed by two-tailed unpaired Student's t-test. Results are shown as mean ± SD. Data representative of 2-3 independent experiments. Source data are provided as a Source Data file.

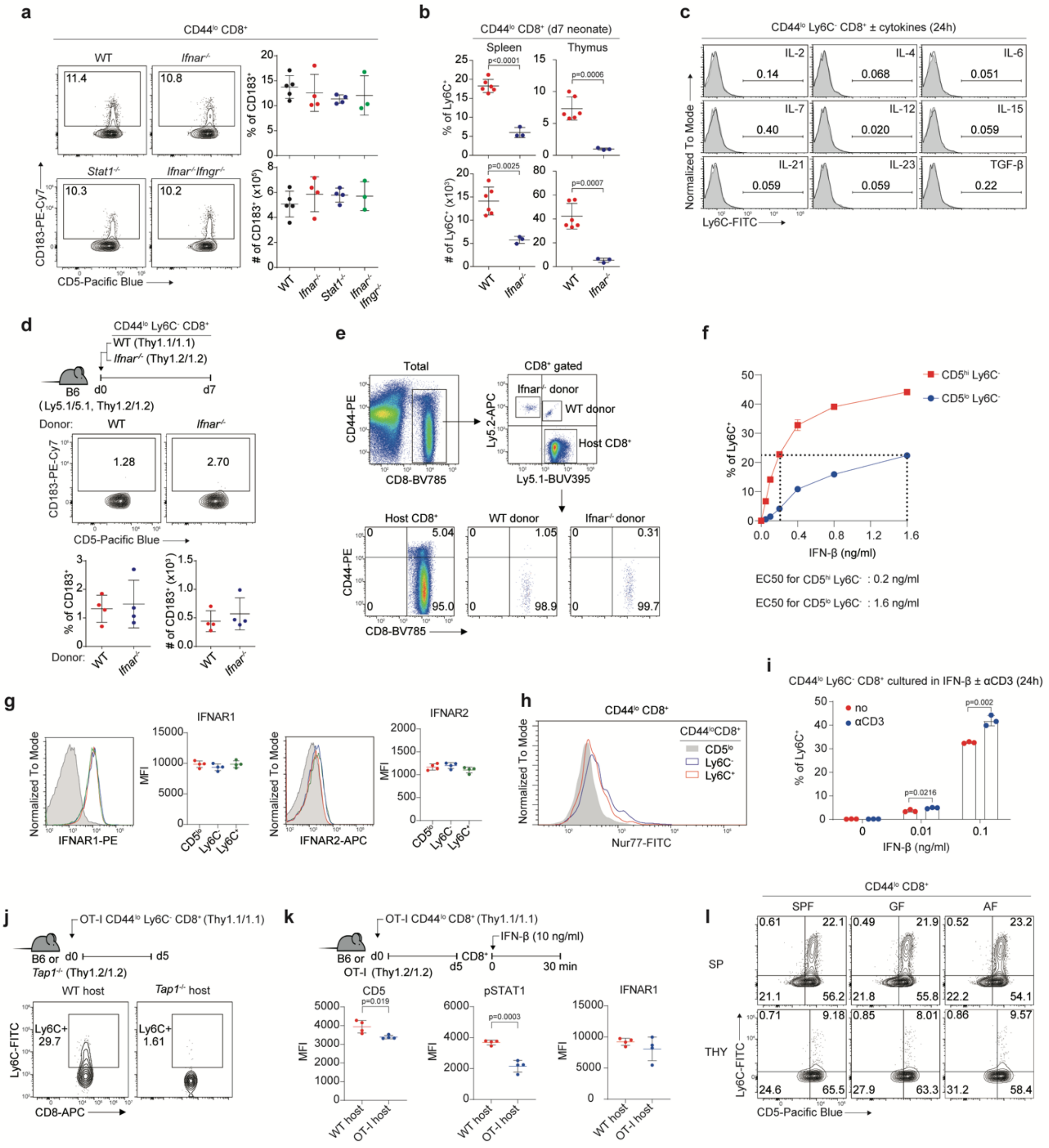

Supplementary Figure 2

Effect of type I IFN on Ly6C, CD183, and CD103 expression in naive CD8$^+$ T cells

a, Percentage and absolute number of CD183$^+$ cells in spleen from different type I IFN signal deficient mice (n=5 for WT, n=4 for *Ifnar*$^{-/-}$, n=4 for *Stat1*$^{-/-}$, n=3 for *Ifnar*$^{-/-}$ *Ifngr*$^{-/-}$ mice). **b,** Percentage and absolute of Ly6C$^+$ cells in spleen and thymus from WT and *Ifnar*$^{-/-}$ neonatal mice (n=6 for WT, n=3 *Ifnar*$^{-/-}$ mice). **c,** Percentage of induced Ly6C$^+$ cells from B6 CD44^{lo}Ly6C$^-$ cells cultured with various cytokines. **d** and **e,** Percentage and absolute of

CD183^{+} CD8^{+} T cells (n=4 mice for each group) (**d**) and CD44 expression profile (**e**) from WT and *Ifnar*$^{-/-}$ CD44^{lo}Ly6C^{-} donor cells. **f,** Percentage of induced Ly6C^{+} cells from B6 CD44^{lo}Ly6C^{-} CD5^{lo} and CD5^{hi} cells cultured with various amounts of IFN-β (n=3 mice for each group). **g** and **h** Expression of IFNAR1 and IFNAR2 (n=4 mice for each group) (**g**) and Nur77 expression (**h**) on CD5^{lo}, Ly6C^{-}, and Ly6C^{+} naive CD8^{+} T cells. **i,** Percentage of induced Ly6C^{+} cells from CD5^{lo} and Ly6C^{-} B6 naive T cells cultured with IFN-β with or without soluble anti-CD3 (n=3 mice for each group). **j,** Percentage of Ly6C^{+} cells induced from Ly6C^{-} naive OT-I CD8^{+} T cells transferred to WT or *Tap1*$^{-/-}$ hosts. **k,** Levels of CD5, phospho-STAT1 and IFNAR1 expression in OT-I donor cells transferred to B6 or OT-I hosts (n=4 mice for each group). **l,** Percentage of naive CD8^{+} T cell subsets from SPF, GF, and AF mice. Statistical significance was confirmed by two-tailed unpaired Student's t-test. Results are shown as mean ± SD. Data representative of 2-3 independent experiments. Source data are provided as a Source Data file.

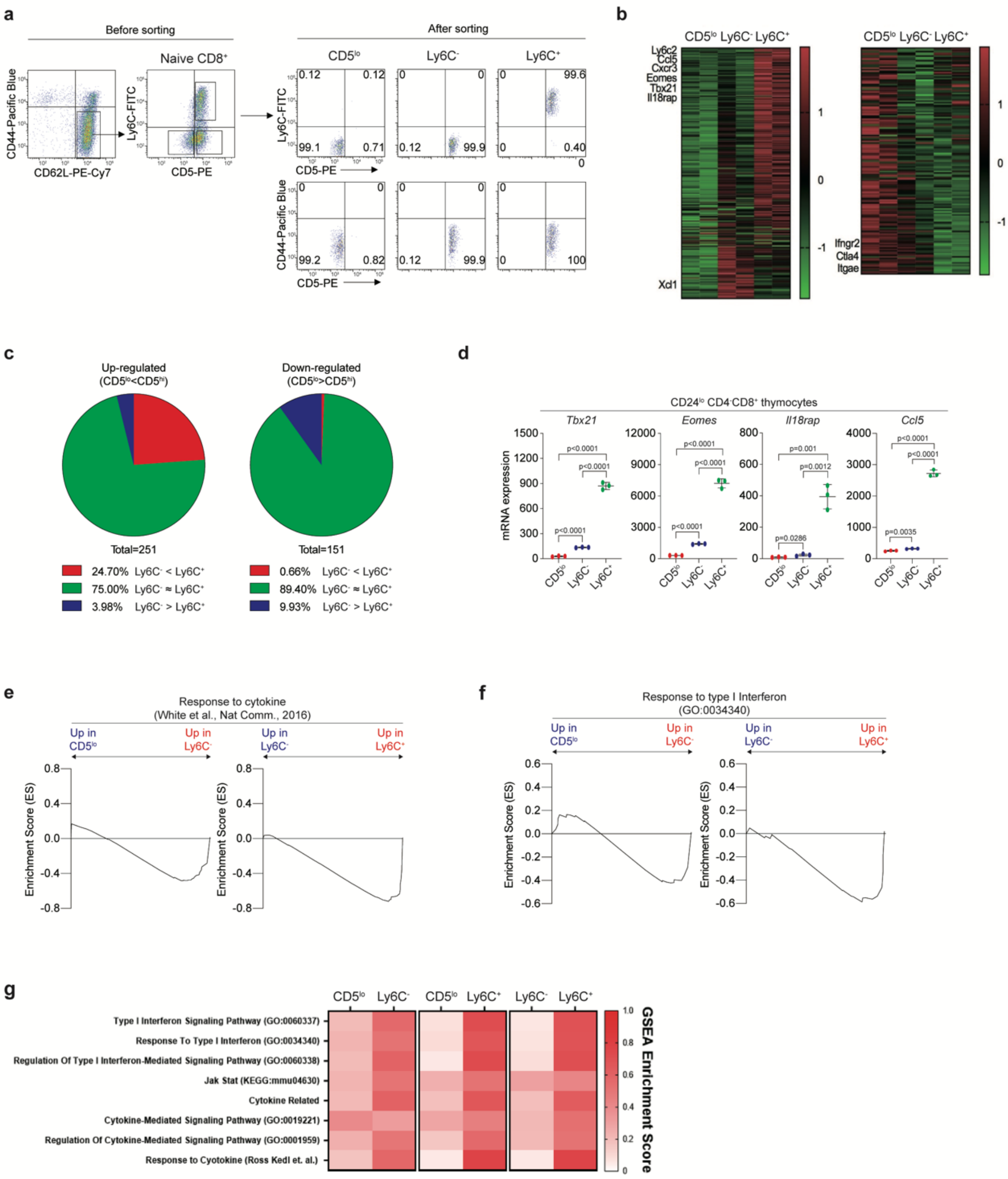

Supplementary Figure 3

Different transcriptomes in distinct subsets of naive CD8$^+$ T cells

a, Gating strategy and purity before and after cell sorting. **b,** Microarray of CD5^{lo}, Ly6C$^-$ and Ly6C$^+$ cells were performed to generate the heat map that shows genes that are either up-regulated (n=328 genes) (left panel) or down- (n=151 genes) (right panel) in CD5^{hi} cells compared to CD5^{lo} cells. The color scale is based on Z-score scaling from -1.75 (Green) to 1.75 (Red). **c,** Pie charts illustrating genes that are regulated similarly or differently in Ly6C$^-$

and Ly6C^{+} cells in up- (left panel) or down-regulated (right panel) genes. **d,** RT-PCR results of *Tbx21, Eomes, Il18rap, and Ccl5* genes in CD5^{lo}, Ly6C^{-} and Ly6C^{+} CD24^{lo}CD4^{-}CD8^{+} thymocytes (n=3 mice for each group). Y-axis represent relative mRNA expression. Statistical significance was confirmed by two-tailed unpaired Student's t-test. Results are shown as mean ± SD. Data representative of two independent experiments. **e** and **f,** GSEA results between CD5^{lo} and Ly6C^{-}, or Ly6C^{-} and Ly6C^{+} cells for pathway: Response to cytokine (White et al., Nat Comm., 2016) (**e**) and Response to type I IFN (GO:0034340) (**f**). **g,** GSEA were performed between CD5^{lo} and Ly6C^{-}, CD5^{lo} and Ly6C^{+}, or Ly6C^{-} and Ly6C^{+} cells for indicated pathways. GSEA Enrichment Score (ES) represent either minimum or maximum running enrichment score. The color scale is based on ES, scaling from 0 (White) to 1.0 (Red). Source data are provided as a Source Data file.

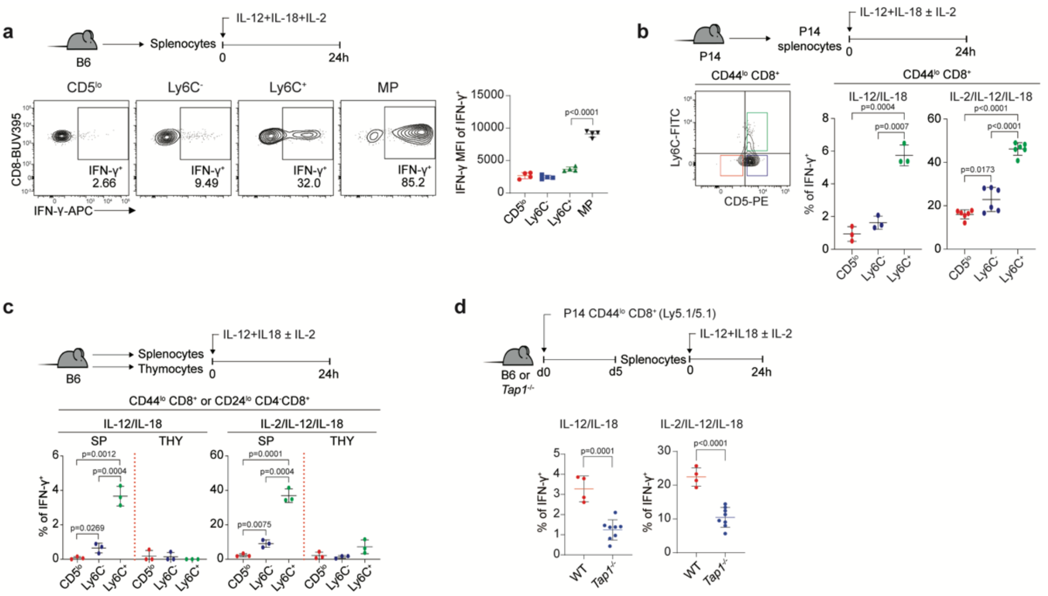

Supplementary Figure 4

Innate function of splenic vs. thymic subsets of naive CD8$^+$ T cells

a, IFN-γ production of B6 naive CD5^{lo}, Ly6C$^-$, and Ly6C$^+$ and MP CD8$^+$ T cells after culture with IL-12/IL-18 (n=4 mice for each group). **b,** IFN-γ production of CD5^{lo}, Ly6C$^-$, and Ly6C$^+$ P14 naive CD8$^+$ T cells after culture with IL-12/IL-18 (n=3 mice for IL-12/IL-18, n=6 mice for IL-2/IL-12/IL-18). **c,** IFN-γ production of CD5^{lo}, Ly6C$^-$, and Ly6C$^+$ cells in CD44^{lo}CD8$^+$ splenocytes and CD24^{lo}CD4$^-$CD8$^+$ thymocytes after culture with IL-12/IL-18 (n=3 mice for each group). **d,** IFN-γ production of P14 naive CD8$^+$ T cells that had been parked for 5 days in either WT or *Tap1*$^{-/-}$ hosts after culture with IL-12/IL-18 (n=4 for WT, n=8 for *Tap1*$^{-/-}$ mice). Statistical significance was confirmed by two-tailed unpaired Student's t-test. Results are shown as mean ± SD. Data representative of 2-3 independent experiments. Source data are provided as a Source Data file.

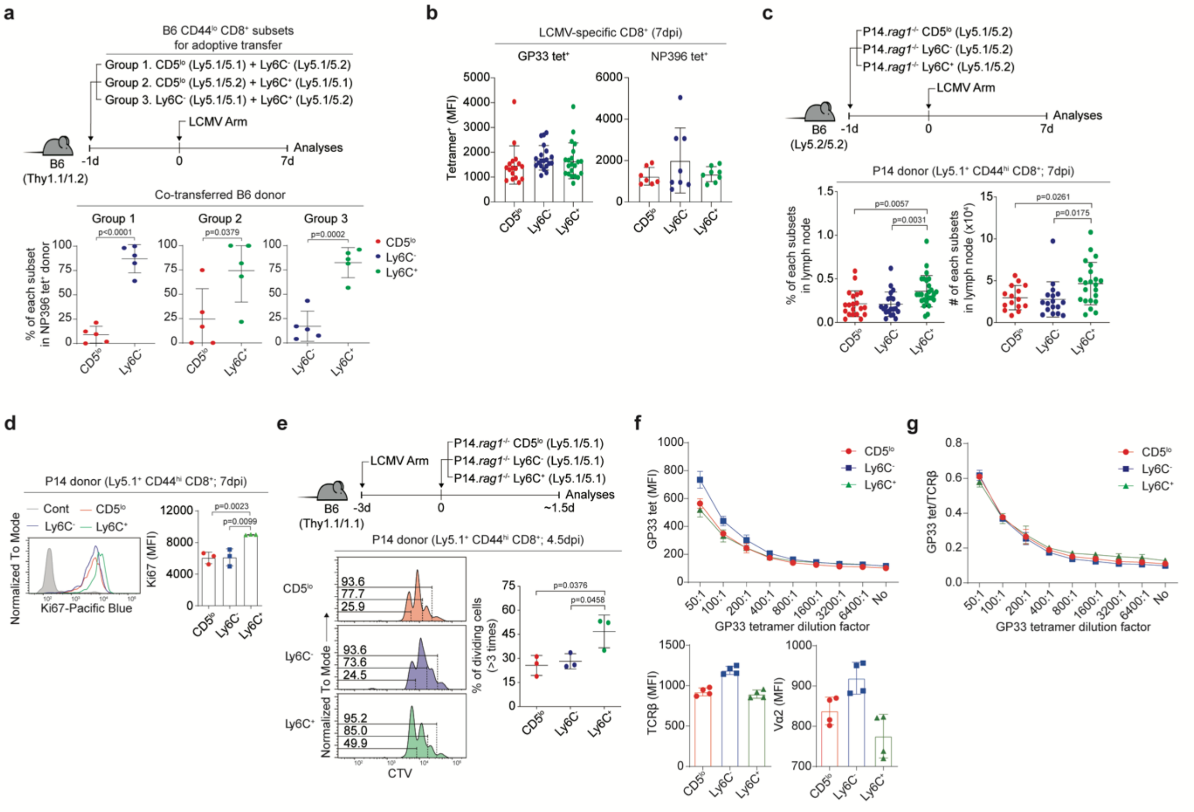

Supplementary Figure 5

The enhanced proliferative responses of Ly6C^{+} cells compared to CD5^{lo} and Ly6C^{-} cells upon LCMV infection

a, Percentage of CD44^{hi}CD8^{+}NP396-tetramer^{+} B6 donor cells (n=5 mice for each group). **b,** Binding intensity of GP33- (left) and NP396- (right) tetramers on CD44^{hi} B6 donor (n=25 mice for GP33 tet^{+}, n=8 mice for NP396 tet^{+}). **c,** Percentage and absolute number of P14 CD44^{hi} donor cells in LN (n=22 mice for each group). **d,** Ki67 expression on P14 CD44^{hi} donor cells (n=3 mice for each group). **e,** CTV dilutions of P14 CD44^{hi} donor cells at 4.5 dpi (n=3 mice for each group). **f,** Binding intensity of GP33-tetramer (upper) and expression of TCRβ (lower left) and Vα2 (lower right) on CD5^{lo}, Ly6C^{-} and Ly6C^{+} P14 naive CD8^{+} T cells (n=4 mice for each group). **g,** Binding affinity of GP33-tetramer normalized by TCRβ expression (n=4 mice for each group). Statistical significance was confirmed by two-tailed unpaired Student’s t-test.

Results are shown as mean ± SD. Data representative of 2-3 independent experiments. Source data are provided as a Source Data file.

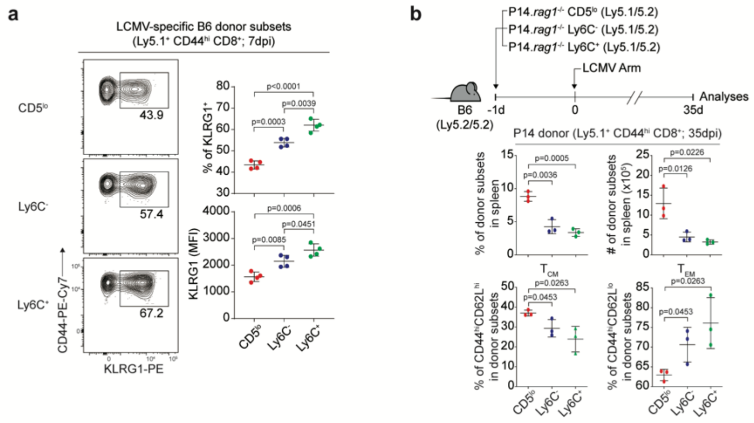

Supplementary Figure 6

Effector and memory differentiation of CD5^{lo}, Ly6C^{-}, and Ly6C^{+} cells upon LCMV infection

a, Percentage of CD44^{hi}KLRG1^{+} donor (n=4 mice for each group). **b,** Percentage and donor recovery of P14 CD44^{hi} donor cells (upper), and percentage of CD44^{hi}CD62L^{hi} and CD44^{hi}CD62L^{lo} P14 donor cells (bottom) at 35 dpi (n=3 mice for each group). Statistical significance was confirmed by two-tailed unpaired Student's t-test. Results are shown as mean ± SD. Data representative of 2-3 independent experiments. Source data are provided as a Source Data file.

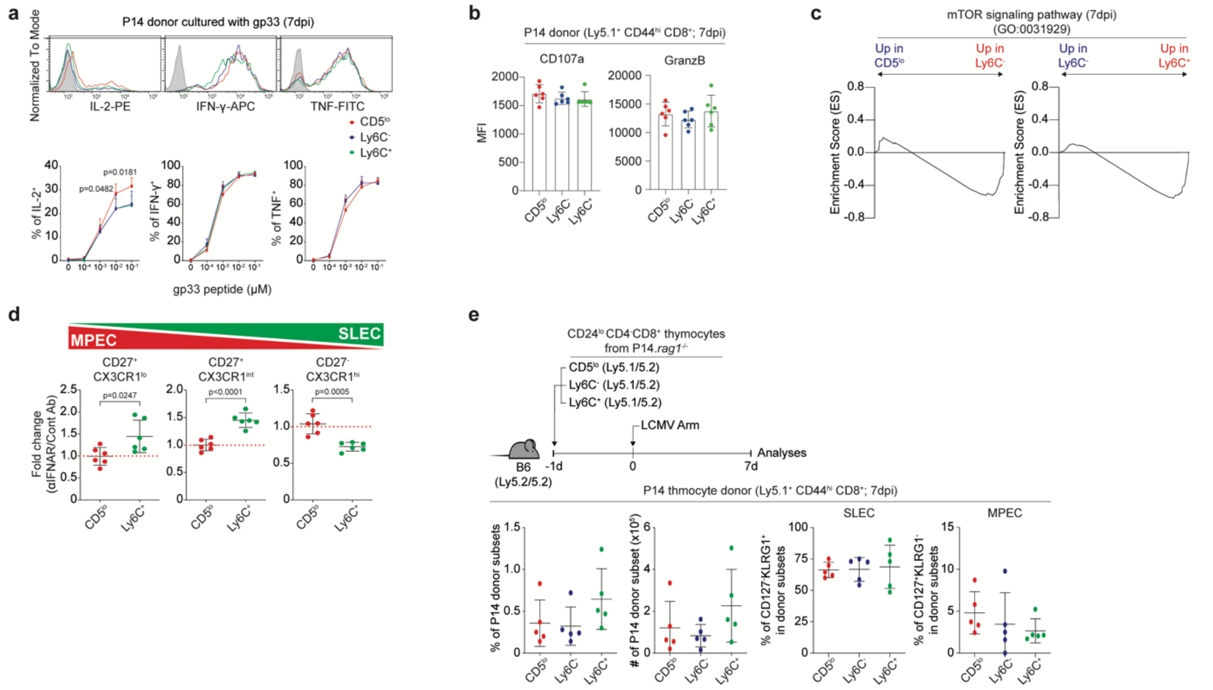

Supplementary Figure 7

Differentiation profiles in splenic and thymic CD5^{lo}, Ly6C$^{–}$, and Ly6C^{+} cells upon LCMV infection

a, IL-2, IFN-γ, and TNF production of P14 CD44^{hi} donor cells stimulated by gp33 peptide (n=6 mice for each group). **b,** CD107a (left) and Granzyme B (right) expression on P14 CD44^{hi} donor cells (n=6 mice for each group). **c,** GSEA results between CD5^{lo} and Ly6C^{+} cells or Ly6C$^{–}$ and Ly6C^{+} P14 CD44^{hi} donor cells for pathway "mTOR signaling pathway". **d,** Fold change of CD27^{+}CX3CR1^{lo}, CD27^{+}CX3CR1^{int}, and CD27$^{–}$CX3CR1^{hi} cells between donor cells previously treated with Control Ab or IFNAR Ab (n=6 mice for each group). **e,** Percentage and donor recovery (left) and percentage of CD127$^{–}$KLRG1^{+} and CD127^{+}KLRG1$^{–}$ cells (right) of P14 thymic donors (n=5 mice for each group). Statistical significance was confirmed by two-tailed unpaired Student's t-test. Results are shown as mean ± SD. Data representative of 2-3 independent experiments. Source data are provided as a Source Data file.

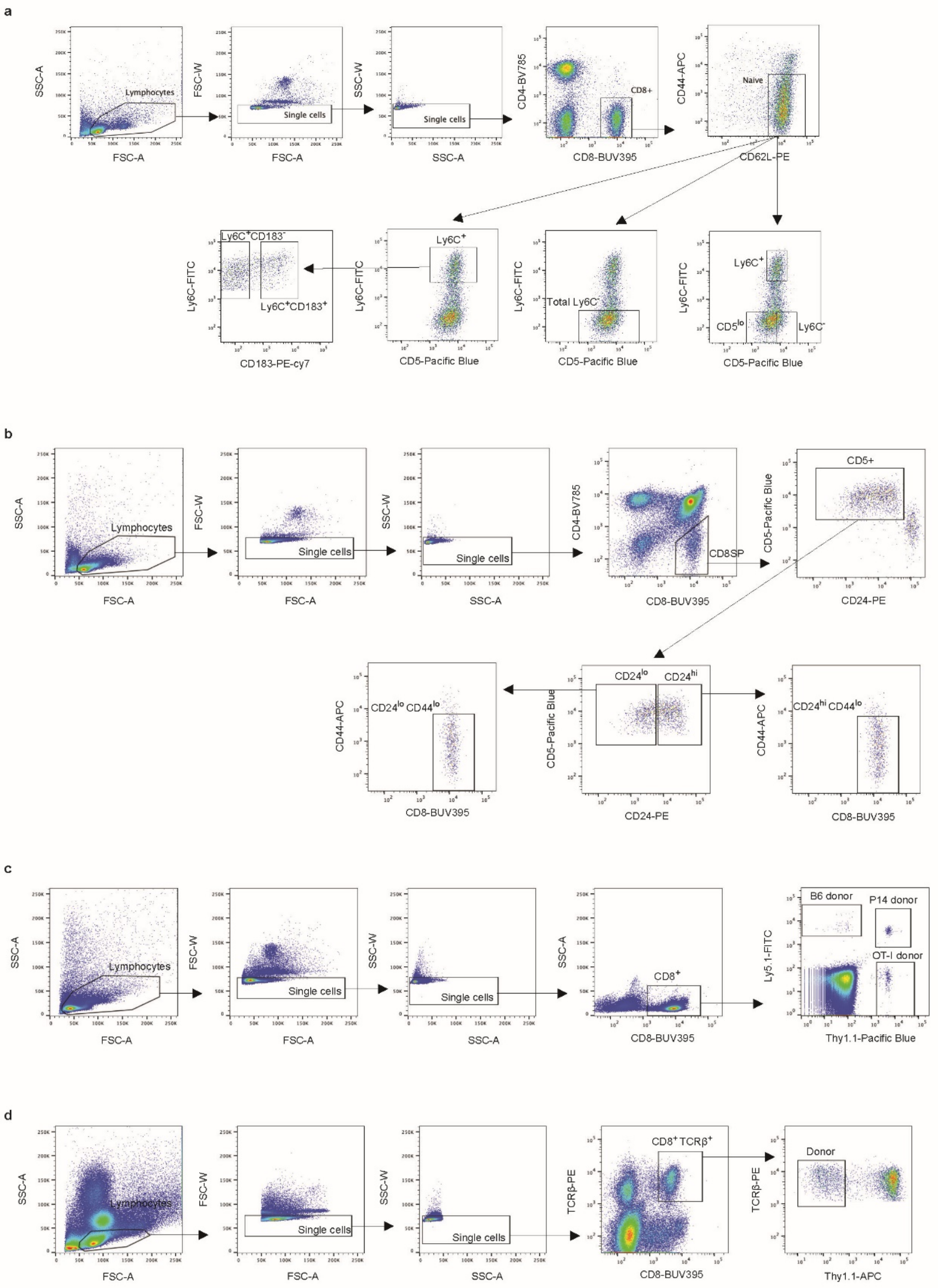
a
SSC-A
FSC-A
Lymphocytes
FSC-W
Single cells
SSC-W
CD4-BV785
CD8-BUV395
CD8+
CD44-APC
CD62L-PE
Naive
Ly6C-FITC
CD183-PE-cy7
Ly6C+CD183-
Ly6C+CD183+
CD5-Pacific Blue
Ly6C+
Total Ly6C-
CD5lo
Ly6C-
b
CD8SP
CD5+
CD24-PE
CD24lo
CD24hi
CD24lo CD44lo
CD24hi CD44lo
c
CD8+
Ly5.1-FITC
Thy1.1-Pacific Blue
B6 donor
P14 donor
OT-I donor
d
TCRβ-PE
CD8+ TCRβ+
Donor
Thy1.1-APC

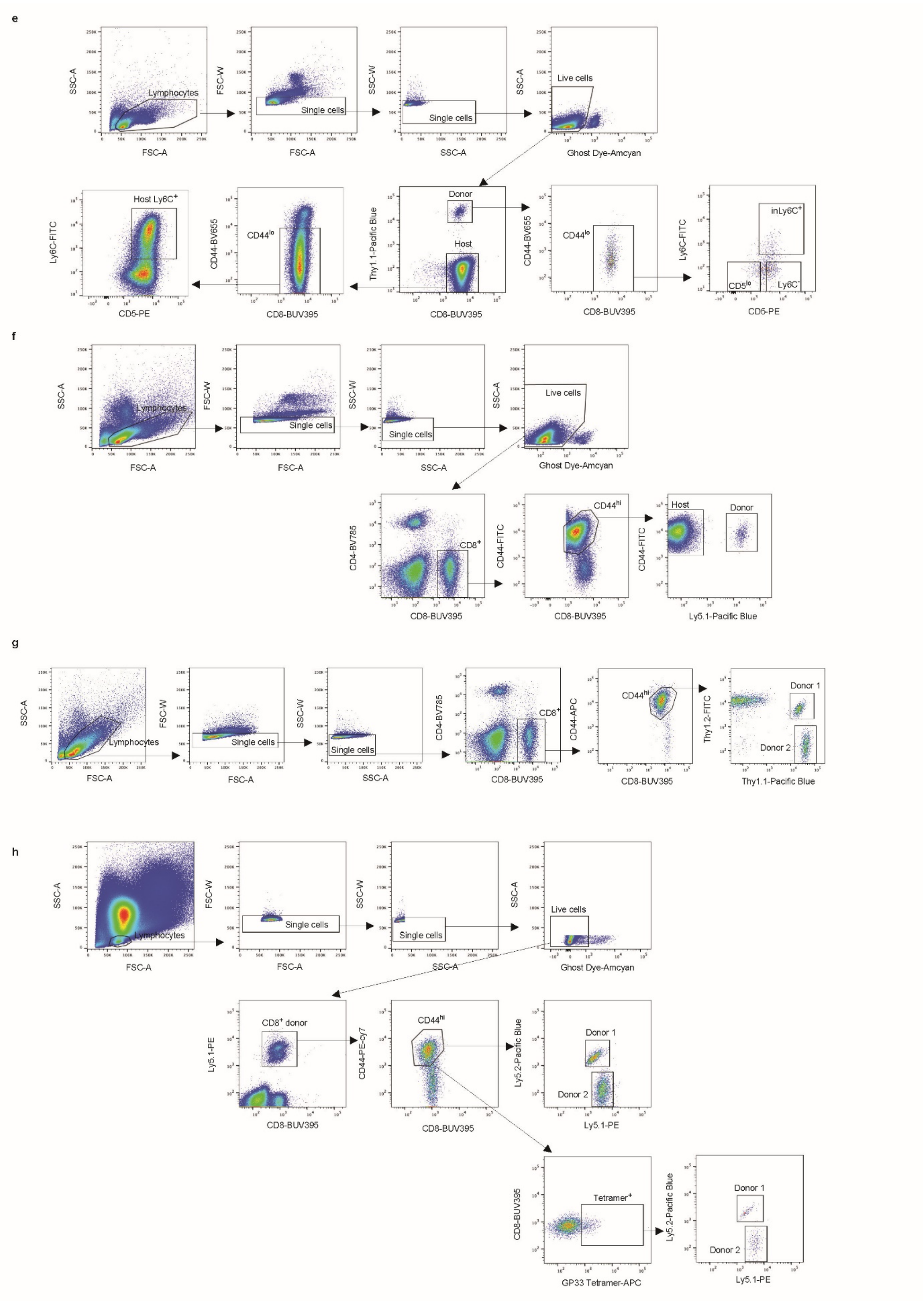

Supplementary Figure 8

Gating strategies

a, Gating strategy for Figures 1b, 1c, 1e, 1f, 2a, 2c, 2e, 2f, 4a, 4b, and 4d. **b,** Gating strategy for Figures 1d, 1e, and 1f. **c,** Gating strategy for Figure 2j. **d,** Gating strategy for Figure 2i. **e,** Gating strategy for Figure 4c. **f,** Gating strategy for Figures 5d, 5e, 5f, 6b, 6c, 6f, 6g, 6h, 7b, and 7c. **g,** Gating strategy for Figures 5b, 5c, 7f, and 7e. **h,** Gating strategy for Figure 5a.

Immgen Cluster	# of genes in cluster	# of core enrichment genes (CD5^{lo} vs Ly6C^{-}, CD5^{lo} vs Ly6C^{+}, Ly6C^{-} vs Ly6C^{+})	Expected # of IFN-β regulated core enrichment genes (CD5^{lo} vs Ly6C^{-}, CD5^{lo} vs Ly6C^{+}, Ly6C^{-} vs Ly6C^{+})	Actual # of IFN-β regulated core enrichment genes (CD5^{lo} vs Ly6C^{-}, CD5^{lo} vs Ly6C^{+}, Ly6C^{-} vs Ly6C^{+})
Cluster I (Initial cytokine or effector response)	16	5, 7, 2	0.51, 0.71, 0.2	$p<0.0001$, $p<0.0001$, $p=0.0104$<br>5, 7, 2
Cluster II (Preparation for cell division)	149	59, 72, 56	6.02, 7.35, 5.72	3, 3, 2
Cluster III (Cell cycle & division)	155	87, 59, 67	8.88, 6.02, 6.84	$p<0.0005$, $p<0.0005$, $p=0.0006$<br>32, 24, 17
Cluster IV (Naive and late memory)	5	3, 2, 3	0.3, 0.2, 0.3	1, 1, 1
Cluster V (Early effector, late memory)	16	12, 14, 13	1.22, 1.43, 1.32	0, 0, 0
Cluster VI (Short-term effector and memory)	34	3, 6, 7	0.3, 0.61, 0.71	$p=0.0059$<br>1, 2, 4
Cluster VII (Memory precursor)	29	10, 7, 7	1.02, 0.71, 0.71	3, 3, 3
Cluster VIII (Naive or late effector or memory)	76	14, 21, 31	1.43, 2.14, 3.16	1, 3, 5
Cluster IX (Short-term effector or memory)	7	3, 4, 2	0.3, 0.4, 0.2	0, 2, 0
Cluster X (Late effector or memory)	16	5, 5, 7	0.51, 0.51, 0.71	1, 1, 1

Supplementary Table 1

Influence of IFN-β on core enrichment genes of Immgen clusters

Influence of IFN-β on core enrichment genes of Immgen clusters between CD5^{lo} and Ly6C$^{–}$, CD5^{lo} and Ly6C^{+}, or Ly6C$^{–}$ and Ly6C^{+} naive B6 CD8^{+} T cells. P-values are calculated using Eq.2 and shown above the values of actual number of IFN-β regulated core enrichment genes.

Name	Company	Clone	Catalogue No.	Application	Dilution
anti-CD103	Biolegend	2E7	121414	Flow cytometry	1:400
anti-CD107a	Biolegend	1D4B	121614	Flow cytometry	1:400
anti-CD122	Biolegend	5H4	105912	Flow cytometry	1:400
anti-CD122	Biolegend	TM-β1	123214	Flow cytometry	1:400
anti-CD123	Biolegend	6H6	306014	Flow cytometry	1:400
anti-CD124	Biolegend	I015F8	144807	Flow cytometry	1:400
anti-CD126	Biolegend	D7715A7	115812	Flow cytometry	1:400
anti-CD127	Biolegend	A7R34	135008	Flow cytometry	1:400
anti-CD130	eBiosciences	KGP130	15536876	Flow cytometry	1:400
anti-CD132	Biolegend	TUGm2	132308	Flow cytometry	1:400
anti-CD183	Biolegend	CXCR3-173	126506	Flow cytometry	1:400
anti-CD24	Biolegend	M1/69	101806	Flow cytometry	1:400
anti-CD25	Invitrogen	PC61.5	16370834	Flow cytometry	1:400
anti-CD27	Biolegend	O323	302806	Flow cytometry	1:400
anti-CD28	Biolegend	37.51	102110	Flow cytometry	1:400
anti-CD38	Biolegend	90	102708	Flow cytometry	1:400
anti-CD3ε	Biolegend	145-2C11	100334	Flow cytometry	1:400
anti-CD4	Biolegend	RM4-5	100510	Flow cytometry	1:400
anti-CD44	Biolegend	IM7	103020	Flow cytometry	1:400
anti-CD45.1	BD Biosciences	A20	565212	Flow cytometry	1:400
anti-CD45.2	Biolegend	104	109820	Flow cytometry	1:400
anti-CD45RA	BD Biosciences	14.8	564360	Flow cytometry	1:400
anti-CD45RB	Biolegend	C363.16A	103305	Flow cytometry	1:400
anti-CD5	Biolegend	53-7.3	100642	Flow cytometry	1:400
anti-CD62L	Biolegend	MEL-14	104408	Flow cytometry	1:400
anti-CD8α	Tonbo	53-6.7	20-0081-U100	Flow cytometry	1:400
anti-CD90.1	eBiosciences	HIS51	15238149	Flow cytometry	1:400
anti-CD90.2	eBiosciences	53-2.1	15298609	Flow cytometry	1:400
anti-CD98	eBiosciences	RL388	15218629	Flow cytometry	1:400
anti-CX3CR1	Biolegend	2A9-1	341610	Flow cytometry	1:400
anti-GITR	Biolegend	DTA-1	126308	Flow cytometry	1:400
anti-Granzyme B	Biolegend	GB11	515406	Flow cytometry	1:200
anti-IFNAR1	SinoBiological	110	50469-R110-P	Flow cytometry	1:400
anti-IFNAR2	R&D Systems	Polyclonal	FAB1083A	Flow cytometry	1:400
anti-IFN-γ	Biolegend	XMG1.2	505814	Flow cytometry	1:200
anti-IL-2	Biolegend	JES6-5H4	503810	Flow cytometry	1:200
anti-Ki-67	eBiosciences	SolA15	15268809	Flow cytometry	1:200
anti-KLRG1	Biolegend	2F1/KLRG1	138412	Flow cytometry	1:400
anti-Ly6C	eBiosciences	HK1.4	15518606	Flow cytometry	1:400
anti-Nur77	Invitrogen	12.14	12-5965-82	Flow cytometry	1:200
anti-TCRβ	Biolegend	H57-597	109226	Flow cytometry	1:400
anti-TNF	Biolegend	MP6-XT22	506314	Flow cytometry	1:200
anti-Vα11.1/11.2	Biolegend	RR8-1	139904	Flow cytometry	1:400
anti-Vα2	eBiosciences	B20.1	15248269	Flow cytometry	1:400
anti-Vα3.2	eBiosciences	RR3-16	15556566	Flow cytometry	1:400
anti-Vα8.3	Biolegend	KT50	125707	Flow cytometry	1:400
anti-Vβ10b	BD Biosciences	B21.5	553284	Flow cytometry	1:400
anti-Vβ11	BD Biosciences	RR3-15	553196	Flow cytometry	1:400
anti-Vβ11.1/11.2	Biolegend	RR8-1	139904	Flow cytometry	1:400
anti-Vβ12	Biolegend	MR11-1	139704	Flow cytometry	1:400
anti-Vβ13	BD Biosciences	MR12-3	561541	Flow cytometry	1:400
anti-Vβ14	BD Biosciences	14-2	553258	Flow cytometry	1:400
anti-Vβ17a	BD Biosciences	KJ23	553212	Flow cytometry	1:400
anti-Vβ2	Biolegend	B20.6	127906	Flow cytometry	1:400
anti-Vβ3	BD Biosciences	KJ25	553208	Flow cytometry	1:400
anti-Vβ4	BD Biosciences	KT4	553365	Flow cytometry	1:400
anti-Vβ5.1/5.2	eBiosciences	MR9-4	16380614	Flow cytometry	1:400
anti-Vβ6	eBiosciences	RR4-7	15300760	Flow cytometry	1:400
anti-Vβ7	Biolegend	TR310	118306	Flow cytometry	1:400
anti-Vβ8	BD Biosciences	F23.1	553861	Flow cytometry	1:400
anti-Vβ8.3	BD Biosciences	1B3.3	553663	Flow cytometry	1:400
anti-Vβ9	BD Biosciences	MR10-2	553201	Flow cytometry	1:400
anti-β2	Biolegend	M18/2	101408	Flow cytometry	1:400
anti-β7	Biolegend	FIB504	321208	Flow cytometry	1:400
GP33-tetramer	Immudex		JA2142-APC	Flow cytometry	1:50
NP396 tetramer	Immudex		JA2160-APC	Flow cytometry	1:50
anti-phospho-STAT1 (Tyr701)	Cell Signaling Technologies	58D6	9167	Western blot	1:1000
anti-phospho-STAT2 (Tyr690)	Cell Signaling Technologies	D3P2P	88410	Western blot	1:1000
anti-b-actin	Sigma-Aldrich	AC-15	A1978-200UL	Western blot	1:5000
m-IgGκ BP-HRP	Santa Cruz Biotechnology		sc-516102	Western blot	1:1000
goat anti-rabbit IgG-HRP	Santa Cruz Biotechnology		sc-2004	Western blot	1:1000

Supplementary Table 2

Antibodies and reagents

Name	Company	Assay ID	Exon Boundary	Amplicon Length	Applications
Ly6C2	Applied Biosystems	Mm00841873_m1	1-2	162	Real-time PCR
Tbx21	Applied Biosystems	Mm00450960_m1	1-2	69	Real-time PCR
Eomes	Applied Biosystems	Mm01351984_m1	1-2	78	Real-time PCR
Il18rap	Applied Biosystems	Mm00516053_m1	9-10	113	Real-time PCR
Ccl5	Applied Biosystems	Mm01302427_m1	1-2	103	Real-time PCR

Supplementary Table 3

Primers
