# Supplementary material for: Self-reactivity controls functional diversity of naive CD8+ T cells by co-opting tonic type I interferon
Source: Nat Commun. 2021 Oct 18;12:6059. doi: 10.1038/s41467-021-26351-3 (PMC8523551; doi:10.1038/s41467-021-26351-3)
Supplement: Supplementary file 3 — Reporting summary [file 41467_2021_26351_MOESM3_ESM.pdf]

nature portfolio

Corresponding author(s): Jae-Ho Cho
Cheol-Heui Yun

Last updated by author(s): Aug 24, 2021

Reporting Summary

Nature Portfolio wishes to improve the reproducibility of the work that we publish. This form provides structure for consistency and transparency in reporting. For further information on Nature Portfolio policies, see our Editorial Policies and the Editorial Policy Checklist.

Statistics

For all statistical analyses, confirm that the following items are present in the figure legend, table legend, main text, or Methods section.

n/a	Confirmed	
☐	☒	The exact sample size (n) for each experimental group/condition, given as a discrete number and unit of measurement
☐	☒	A statement on whether measurements were taken from distinct samples or whether the same sample was measured repeatedly
☐	☒	The statistical test(s) used AND whether they are one- or two-sided<br>*Only common tests should be described solely by name; describe more complex techniques in the Methods section.*
☒	☐	A description of all covariates tested
☒	☐	A description of any assumptions or corrections, such as tests of normality and adjustment for multiple comparisons
☐	☒	A full description of the statistical parameters including central tendency (e.g. means) or other basic estimates (e.g. regression coefficient) AND variation (e.g. standard deviation) or associated estimates of uncertainty (e.g. confidence intervals)
☐	☒	For null hypothesis testing, the test statistic (e.g. F, t, r) with confidence intervals, effect sizes, degrees of freedom and P value noted<br>*Give P values as exact values whenever suitable.*
☒	☐	For Bayesian analysis, information on the choice of priors and Markov chain Monte Carlo settings
☒	☐	For hierarchical and complex designs, identification of the appropriate level for tests and full reporting of outcomes
☒	☐	Estimates of effect sizes (e.g. Cohen's d, Pearson's r), indicating how they were calculated

Our web collection on statistics for biologists contains articles on many of the points above.

Software and code

Policy information about availability of computer code

Data collection	Flow cytometry - BD Bioscience FACSDiva 7.0<br>Quantitative Real-time PCR - StepOne Software 2.2.2<br>Metabolic Assay - Seahorse Wave Controller Software 2.4.2
Data analysis	TreeStar FlowJo 10.6.2<br>GraphPad Prism 8.4.1<br>Broad Institute Gene Set Enrichment Analysis version 4.0.1<br>Seahorse Wave Desktop version 2.2.0.276

For manuscripts utilizing custom algorithms or software that are central to the research but not yet described in published literature, software must be made available to editors and reviewers. We strongly encourage code deposition in a community repository (e.g. GitHub). See the Nature Portfolio guidelines for submitting code & software for further information.

Data

Policy information about availability of data

All manuscripts must include a data availability statement. This statement should provide the following information, where applicable:

- Accession codes, unique identifiers, or web links for publicly available datasets
- A description of any restrictions on data availability
- For clinical datasets or third party data, please ensure that the statement adheres to our policy

RNA-Seq data and Microarray data have been deposited in the NCBI's Gene Expression Omnibus (GEO) database under the primary accession numbers GSE178445,

GSE178447, GSE178448, and GSE178449. The datasets generated during the current study are available from the corresponding author (JHC) upon request. The authors declare that all data supporting the findings of this study are available within the paper. Source data are provided with this paper.

Field-specific reporting

Please select the one below that is the best fit for your research. If you are not sure, read the appropriate sections before making your selection.

☒ Life sciences ☐ Behavioural & social sciences ☐ Ecological, evolutionary & environmental sciences

For a reference copy of the document with all sections, see nature.com/documents/nr-reporting-summary-flat.pdf

Life sciences study design

All studies must disclose on these points even when the disclosure is negative.

Sample size	Group sizes were determined on the basis of preliminary experiments and were found sufficient to reveal biologically relevant differences among the samples of interest.
Data exclusions	No data were excluded from the analyses.
Replication	All experiments were repeated at least twice and and gave comparable results each time.
Randomization	Mice were allocated to experimental groups based on sex and age matched within experiments.
Blinding	Investigators were not blinded to group allocation. Since most experiments were done by one investigator, the investigator needed to know the exact condition of the experimenting mice. To avoid bias of the investigator, all mice were bred and experimented on the same day with the same procedures, analysis were carried out with authorized softwares using strict standards (e.g. gating strategy). Therefore, no blinding was necessary.

Reporting for specific materials, systems and methods

We require information from authors about some types of materials, experimental systems and methods used in many studies. Here, indicate whether each material, system or method listed is relevant to your study. If you are not sure if a list item applies to your research, read the appropriate section before selecting a response.

Materials & experimental systems

n/a	Involved in the study	
☐	☒	Antibodies
☒	☐	Eukaryotic cell lines
☒	☐	Palaeontology and archaeology
☐	☒	Animals and other organisms
☒	☐	Human research participants
☒	☐	Clinical data
☒	☐	Dual use research of concern

Methods

n/a	Involved in the study	
☒	☐	ChIP-seq
☐	☒	Flow cytometry
☒	☐	MRI-based neuroimaging

Antibodies

Antibodies used

Antibody (clone), Manufacturer, Cat#

anti-CD45RA (HI100), Biolegend, 304108
anti-CD45RB (C363.16A), Biolegend, 103308
anti-CD124 (X2/45-12), eBioscience, 14-1249-82
anti-Vα2 (B20.1), BD Pharmagen, 553288
anti-Vα3.2 (RR3-16), Biolegend, 135404
anti-Vα8.3 (KT50), Biolegend, 125707
anti-Vα11.1/11.2 (RR8-1), Biolegend, 139904
anti-Vβ2 (B20.6), Biolegend, 127906
anti-Vβ3 (KJ25), BD Pharmagen, 553208
anti-Vβ4 (KT4), BD Pharmagen, 553365
anti-Vβ5.1/5.2 (MR9-4), BD Pharmagen, 562087
anti-Vβ6 (RR4-7), BD Pharmagen, 553193
anti-Vβ7 (TR310), BD Pharmagen, 553215
anti-Vβ8 (F23.1), BD Pharmagen, 553861
anti-Vβ8.3 (1B3.3), BD Pharmagen, 553663
anti-Vβ9 (MR10-2), BD Pharmagen, 553201
anti-Vβ10 (B21.5), BD Pharmagen, 553284

anti-Vβ10b (B21.5), BD Pharmagen, 553284
anti-Vβ11 (RR3-15), BD Pharmagen, 553197
anti-Vβ11.1/11.2 (RR8-1), BD Pharmagen, 553222
anti-Vβ12 (MR11-1), BD Pharmagen, 553300
anti-Vβ13 (MR12-3), BD Pharmagen, 553204
anti-Vβ14 (14-2), BD Pharmagen, 553258
anti-Vβ17a (KJ23), BD Pharmagen, 553212
anti-Granzyme B (GB11), BD Pharmagen, 560211
anti-CD107a (1D4B), BD Pharmagen, 558661
anti-CD3ε (145-2C11), Biolegend, 100312
anti-CD4 (RM4-5), Biolegend, 100530
anti-CD5 (53-7.3), Biolegend, 100618
anti-CD25 (PC61.5), BD Pharmagen, 557658
anti-CD27 (O323), Biolegend, 302806
anti-CD28 (37.51), Biolegend, 102110
anti-CD38 (90), Biolegend, 102712
anti-CD44 (IM7), Biolegend, 103030
anti-CD103 (2E7), Biolegend, 121422
anti-CX3CR1 (2A9-1), Biolegend, 341610
anti-IFN-γ (XMG1.2), Biolegend, 505830
anti-IL-2 (JES6-5H4), Biolegend, 503810
anti-TNF-α (MP6-XT22), Biolegend, 506313
anti-CD62L (MEL-14), Biolegend, 104421
anti-CD24 (1M/69), Biolegend, 101806
anti-CD45.1 (A20), Biolegend, 110743
anti-CD45.2 (104), Biolegend, 109832
anti-CD90 (5E10), Biolegend, 328108
anti-CD90.1 (HIS51), eBioscience, 17-0900-82
anti-CD90.2 (53-2.1), eBioscience, 12-0902-82
anti-CD98 (RL388), Biolegend, 128208
anti-CD122 (5H4), Biolegend, 105912
anti-CD122 (TM-β1), Biolegend, 123214
anti-CD123 (6H6), Biolegend, 306012
anti-CD126 (D7715A7), Biolegend, 115812
anti-CD127 (A7R34), Biolegend, 135010
anti-CD130 (KGP130), eBioscience, 17-1302-82
anti-CD132 (4G3), BD Pharmagen, 554457
anti-CD183 (CXCR3-173), Biolegend, 126516
anti-Ly6C (HK1.4), eBioscience, 17-5932-82
anti-TCRβ (H57-597), Biolegend, 109212
anti-β2 (TS1/18), eBioscience, MA1810
anti-β7 (FIB504), Biolegend, 321204
anti-GITR (DTA-1), Biolegend, 126308
anti-KLRG1 (2F1), Biolegend, 138408
anti-Ki-67 (SolA15), eBioscience, 11-5698-82
anti-Nur77 (12.14), eBioscience, 12-5965-82
anti-IFNAR1 (110), SinoBiological, 50469-R110-P
anti-CD8α (53-6.7), Tonbo, 20-0081
anti-phospho-STAT1 (Tyr701) (58D6), Cell Signaling Technology, 9167
anti-phospho-STAT2 (Tyr690) (D3P2P), Cell Signaling Technology, 88410
anti-b-actin (AC-15), Sigma-Aldrich, A1978
GP33-tetramer, Immudex, JA2160
NP396 tetramer, Immudex, JA2142
m-IgGκ BP-HRP, Santa Cruz Biotechnology, sc-516102
goat anti-rabbit IgG-HRP, Santa Cruz Biotechnology, sc-2004
anti-IFN a/b R2 (polyclonal), R&D Systems, FAB1083A
anti-BrdU (BU20A), invitrogen, 11-5071-42

Validation

All antibodies used are commercially available, and were validated with mouse for western blot(anti-phospho-STAT1, anti-phospho-STAT2, anti-b-actin, m-IgGk BP-HRP, goat anti-rabbit IgG-HRP) and flow cytometry (all the antibodies except for those used for western blot) application by the manufactures. Validation statement for each antibody is provided on manufacture's websites.

Animals and other organisms

Policy information about studies involving animals; ARRIVE guidelines recommended for reporting animal research

Laboratory animals

C57BL/6 (B6), B6.SJL (Ly5.1) and B6.PL (Thy1.1) mice were purchased from The Jackson Laboratory.
P14, OT-I Thy1.1, Tap1–/–, Rag1–/–, Ifnar–/–, Ifnar.Ifngr–/–, and Stat1–/– mice (all on a B6 background) were obtained from POSTECH.
P14.Rag1–/– Ly5.1 and P14.Rag1–/– Thy1.1 mice were generated by crossing P14 mice with Rag1–/– and B6.SJL or B6.PL mice.
Mice were maintained under specific pathogen-free conditions.
Germ-free (GF) and antigen-free (AF) mice are maintained sterilely at POSTECH Biotech Center.
Unless described otherwise, all mice were used sex-matched at 8-12 weeks of age, according to protocols approved by the Animal Experimental and Ethic Committee at POSTECH and Chonnam National University (CNU).
For experiment, only the female mice (in all strains) were used.

Wild animals	This study did not use wild animals.
Field-collected samples	This study did not use field-collected samples.
Ethics oversight	All experiment utilizing mice were conducted and performed according to protocols approved by the Animal Experimental and Ethic Committee at POSTECH and Chonnam National University (CNU).

Note that full information on the approval of the study protocol must also be provided in the manuscript.

Flow Cytometry

Plots

Confirm that:

☒ The axis labels state the marker and fluorochrome used (e.g. CD4-FITC).

☒ The axis scales are clearly visible. Include numbers along axes only for bottom left plot of group (a 'group' is an analysis of identical markers).

☒ All plots are contour plots with outliers or pseudocolor plots.

☒ A numerical value for number of cells or percentage (with statistics) is provided.

Methodology

Sample preparation	Isolated mouse LN and spleen suspensions were prepared by gently pressing LN and spleen trough 70µm cell strainer and washed with media. In case of spleen, to remove red blood cells, the suspension was resuspended with RBC lysis buffer for 3 min on ice, and wash with media again. The isolated single cells were prepared and stained with flourochrome-conjugated antibodies appropriating to each experimental design indicated on method section and legends.
Instrument	Samples were analyzed by FACS Canto II and LSR II (BD). Cell sorting was performed by utilizing MoFlo Astrios or XDP (Beckman coulter)
Software	Data was collected utilizing FACSDiva software (BD) and analyzed by FlowJo (TreeStar). Statistics of data were conducted by Prism (GraphPad).
Cell population abundance	Cell subsets were sorted with >98% purity as controlled by remeasurement of sorted populations.
Gating strategy	Lympocytes were initially gated by FSC(A)/SSC(A), followed by FSC(W/H) to exclude cell doublets. Also dead cells were gated out by utilizing with viability dye before further gating for analyses. Further detail gating strategies were indicated on each figure and legend.

☒ Tick this box to confirm that a figure exemplifying the gating strategy is provided in the Supplementary Information.
